# Key lessons from the COVID-19 pandemic: Role of intensive care, politics and science communication (Review)

**DOI:** 10.3892/mi.2025.259

**Published:** 2025-08-08

**Authors:** Ioannis N. Mammas, Michalis Agrafiotis, Aikaterini Kazani, Chryssie Koutsaftiki, Alexia Papatheodoropoulou, Simon B. Drysdale, Maria Theodoridou, Demetrios A. Spandidos

**Affiliations:** 1 Laboratory of Clinical Virology, Medical School, University of Crete, 71003 Heraklion, Greece; 2 First Department of Paediatrics, University of Athens School of Medicine, 11527 Athens, Greece; 3 Paediatric Clinic, Aliveri 34500, Euboea, Greece; 4 Cardiothoracic Surgery Intensive Care Unit (ICU), ‘Georgios Papanikolaou’ General Hospital of Thessaloniki, Exochi, 57010 Thessaloniki, Greece; 5 Department of Obstetrics and Gynaecology, General Hospital of Chalkida, Chalkida 34100, Euboea, Greece; 6 COVID-19 Reference Centre, ‘Rafina’ Health Care Centre, 19009 Rafina, Greece; 7 Paediatric Intensive Care Unit (PICU), University Hospital of Patras, 26504 Rio, Greece; 8 Oxford Vaccine Group, Department of Paediatrics, University of Oxford, OX3 9DU Oxford, UK; 9 The NIHR Oxford Biomedical Research Centre, OX3 9DU Oxford, UK

**Keywords:** COVID-19, intensive care, high flow nasal oxygen therapy, politics, science communication

## Abstract

The post-coronavirus disease 2019 (COVID-19) era calls for a comprehensive analysis of the recent COVID-19 pandemic to extract important lessons for the international scientific community for the improvement of its readiness towards future pandemic threats and challenges. The present review article presents key aspects of the COVID-19 pandemic, with the main topics covered being the following: i) The recent advances in intensive care, focusing particularly on high flow nasal oxygen therapy; ii) COVID-19 and politics; and iii) COVID-19 and science communication. Both medical aspects of the COVID-19 pandemic as well as non-medical issues, including politics and science communication, should be further evaluated and be definitely included in future medical educational programs, worldwide.

## 1. Introduction

**Figure 1:**
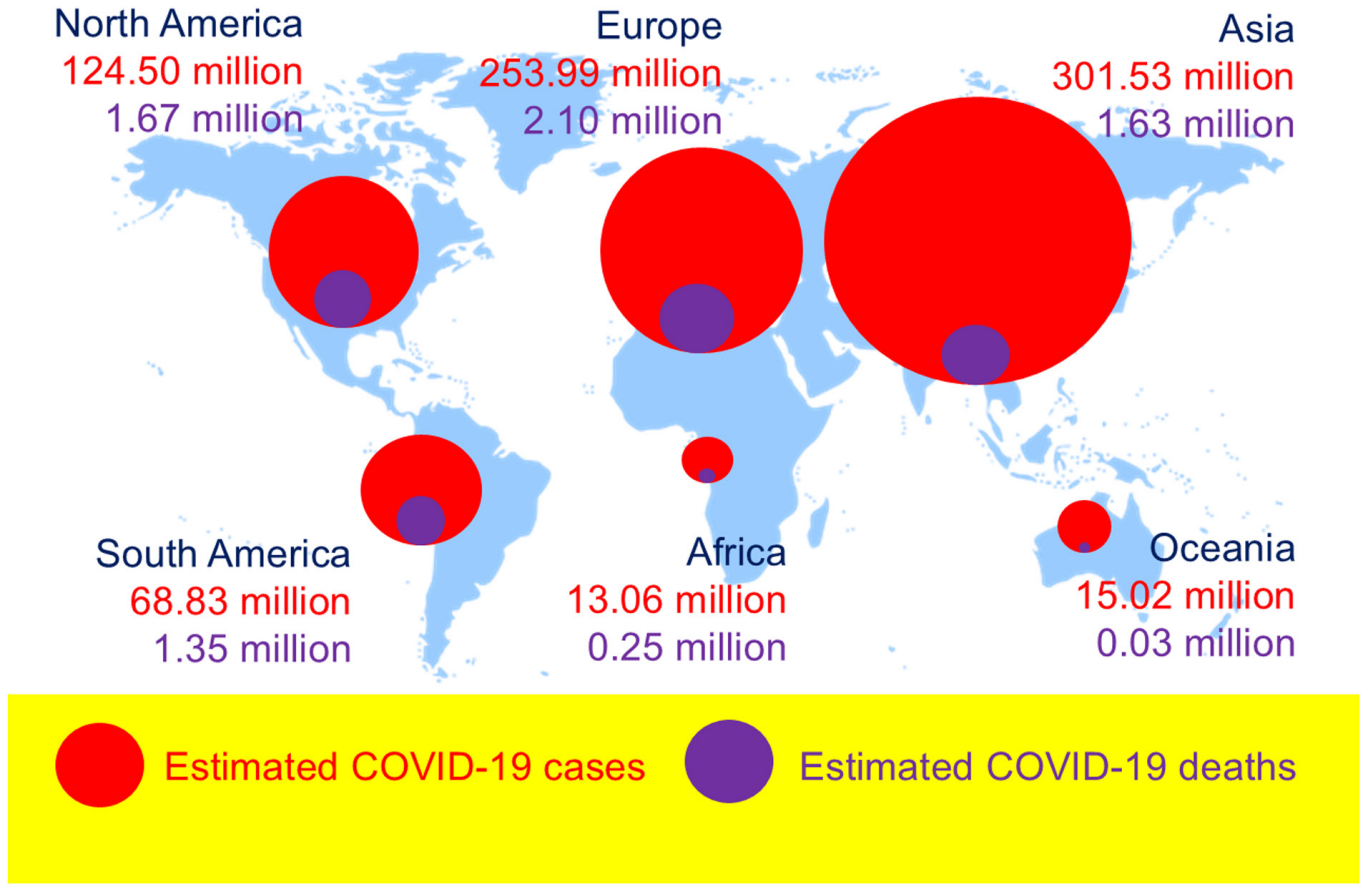
Global distribution of SARS-CoV-2. Confirmed COVID-19 cases and deaths up to 2025, as estimated by the World Health Organization (WHO), by world continent (North America, South America, Europe, Asia and Oceania). SARS-CoV-2, severe acute respiratory syndrome coronavirus 2; COVID-19, coronavirus disease 2019.

The level of preparedness of the scientific community for the next pandemic remains a critical concern. The ways in which the international scientific community can contribute to minimizing the public health impact of a new pandemic require careful consideration. The evaluation of the recent coronavirus disease 2019 (COVID-19) pandemic is indeed crucial (1-6). Belonging to the broad family of coronaviruses, a well-known family of viruses to the paediatric population, severe acute respiratory syndrome coronavirus 2 (SARS-CoV-2), a positive-sense single-stranded RNA (+ssRNA) virus, emerged as one of the most dangerous pathogens in human history (2). The simple structure of the virus, typical of RNA viruses, such as influenza viruses, human immunodeficiency virus (HIV) and cancer-associated viruses, hindered the ability of the immune system to identify its invasion (2,7). In addition, global genetic variations influenced morbidity and mortality rates related to COVID-19(2). The global distribution of SARS-CoV-2, which since the end of 2019 spread immediately around the globe, causing an enormous health and economic catastrophe (https://ourworldindata.org/grapher/cumulative-covid-cases-region and https://ourworldindata.org/grapher/cumulative-covid-deaths-region) is presented in Fig. 1.

From the very beginning of the pandemic, there was a critical need to develop secure, reliable and effective vaccines and therapeutic agents against SARS-CoV-2 (2,8). Lockdown and social distancing significantly influenced social and behavioral aspects of human life. Concurrently, COVID-19 affected the prevalence of other diseases, including cardiovascular diseases and cancer, while in tropical and subtropical areas of the world, COVID-19 lockdown resulted in a significant reduction in the rates of other infections, such as the Dengue fever (9). The mutations of SARS-CoV-2 resulted in the continuing emergence of new COVID-19 cases in all age groups, including children, for several months (7). SARS-CoV-2 infection remains prevalent, while efforts to fund research on COVID-19 have continued to the present day. Research has also focused on post-COVID-19 syndrome and its management (10).

Medical advances and improvements, as well as management limitations, weaknesses and challenges, encountered during the COVID-19 pandemic, require an up-to-date evaluation. In addition to the medical aspects of the pandemic and issues pertaining to strategic preparedness and response planning (1-5), it is also important to systematically analyze non-medical issues, including politics and science communication. The lessons derived from this evaluation will help the prioritisation of research and strategic planning in the event of a future pandemic. Moreover, this analysis will guide the development of up-to-date educational programs to be integrated in both undergraduate and postgraduate medical training, worldwide.

The purpose of the present review article is to summarize the key messages on the lessons learnt from the recent COVID-19 pandemic, one of the most critical and disruptive events of modern times. The main topics on COVID-19 that are discussed herein are: i) Advances in intensive medicine during the COVID-19 pandemic, focusing on high flow nasal oxygen therapy (HFNOT); ii) COVID-19 and politics; and iii) COVID-19 and science communication (Table I).

## 2. High flow nasal oxygen therapy: Not just another oxygen delivering modality

During the recent COVID-19 pandemic, intensive care medicine came to the forefront of the fight against SARS-CoV-2. Thus far, the learning experience has been intense and has affected every aspect of this medical specialty, from therapeutic tools to management strategies and protocols. HFNOT is a relatively novel method for delivering warm humidified oxygen at high flows to patients with acute hypoxaemic respiratory failure (11). The interest in HFNOT and its potential has been further increased during the COVID-19 pandemic, which imposed significant demands on hospital resources, necessitating prudent patient prioritization and careful allocation of respiratory care equipment and intensive care unit (ICU) beds (12).

The HFNOT system setup is simple: It requires only a flow generator, an active heated humidifier, a single heated circuit with a servo-controlled heating wire, and a silicone nasal cannula (11). HFNOT has emerged as an effective and well-tolerated respiratory support technique in various clinical scenarios, although the optimal method for managing acute hypoxaemic respiratory failure remains under debate. Physiological studies have demonstrated that HFNOT, apart from being an effective oxygenator, reduces the work of breathing and respiratory resistance, increases positive end-expiratory pressure and end-inspiratory lung volume, washes off anatomic dead space and improves secretion clearance (12). A common practice is to start HFNOT with a fraction of inspired oxygen (FiO_2_) of 100% and a flow of 60 l/min and then adjust FiO_2_ and the flow to achieve an oxygen saturation (SpO_2_) >88-90% and an age-appropriate respiratory rate (RR) (13). A ROX index (calculated as the SpO_2_/FiO_2_ ratio divided by the RR of the patient) >4.88 at 12 h has a high positive predictive value (89.4%) in predicting treatment success (14). The only absolute contraindication for HFNOT is any indication for invasive mechanical ventilation including shock, respiratory and cardiac arrest, bradycardia, severe arrhythmias and an impaired level of consciousness. Facial erythema, skin breakdown and barotrauma may occur in HFNOT users, although these represent less common complications compared with non-invasive ventilation (NIV). Overall, HFNOT is better tolerated than NIV (15).

Prior to the COVID-19 era, Hernández *et al* (16,17) demonstrated that HFNOT compared with conventional oxygen therapy (COT) decreases the risk of reintubation and post-extubation respiratory failure in ‘low-risk’ ICU patients, while in ‘high-risk’ patients HFNOT was not inferior to NIV in averting reintubation and post-extubation respiratory failure. However, neither of these two studies (16,17) noted any benefit in terms of mortality rates. In a meta-analysis by Zhu *et al* (18) that followed, HFNOT reduced the risk of post-extubation respiratory failure, improved oxygenation and reduced respiratory rates in post-extubated ICU patients. Moreover, in another meta-analysis by Granton *et al* (19), HFNOT reduced re-intubation rates compared with COT, but not when compared with NIV. However, other researchers have failed to duplicate these findings (20). Thus, in another meta-analysis by Maitra *et al* (21) comparing HFNOT with NIV and COT in patients with acute hypoxaemic respiratory failure, no benefit was shown for HFNOT in decreasing requirements for higher respiratory support. Nevertheless, more recently Seow *et al* (22) reviewed a total of 63 studies [including 23 randomized controlled trials (RCTs)], which compared HFNOT with COT and showed that HFNOT decreased the risk for escalating to NIV or invasive respiratory support. In the paediatric population, several RCTs have suggested that compared with COT, HFNOT reduced the rates of intubation and mechanical ventilation in children with moderate-to-severe bronchiolitis and hypoxaemic respiratory failure (23-26). HFNOT is a growing respiratory treatment for children, particularly for those with respiratory distress, bronchiolitis, or other respiratory illnesses.

Focusing on acute hypoxaemic respiratory failure in patients with COVID-19, a meta-analysis of 40 studies including two RCTs by Arruda *et al* (27), suggested that HFNOT reduced the risk of intubation compared with COT, but showed no additional benefit when compared with NIV. In another meta-analysis by Li *et al* (28), again focusing on patients with COVID-19, HFNOT was demonstrated to reduce the rate of intubation, 28-day mortality and ventilator-free days compared with COT. However, these results were not reproduced by a recent meta-analysis by Pisciotta *et al* (29) involving patients with COVID-19-induced hypoxaemic respiratory failure, which showed no benefit in terms of treatment failure for HFNOT compared with NIV and COT.

Recent guidelines issued by the European Respiratory Society (ERS) suggest HFNOT over NIV or COT for the management of acute hypoxaemic respiratory failure. However, although they favor HFNOT over COT for post-extubated ICU patients with ‘low-’ or ‘moderate-risk’ for re-intubation, they suggest NIV over HFNOT for ‘high-risk’ patients (30). During the COVID-19 pandemic, HFNC was also widely applied in the early management of hypoxemia and respiratory distress in children with COVID-19 requiring paediatric intensive care (31,32).

## 3. COVID-19 and politics: Challenges, dilemmas and lessons

Politics was one of the most significant, non-medical issues of the recent COVID-19 pandemic, which demonstrated the interactions between science, society and politics (33). Since the onset of this unprecedented global health challenge, numerous countries designed and implemented various and controversial policies against SARS-CoV-2(34). For example, the ‘zero-COVID-19 policy’, which was adopted by China as well as other countries, tried strictly to eliminate local transmission of the virus (35-37). On the other hand, the ‘Swedish COVID-19 approach’, which did not enforce strict lockdown measures, was based on voluntary recommendations and guidelines (38). Concurrently, Latin American countries appeared to struggle with implementing specific COVID-19 pandemic policies for their citizens (39).

Politics influenced the development, distribution and access of vaccines and therapeutic agents against SARS-CoV-2, as well as public health management and social reaction. The accomplishment of this task would not have been possible without the close collaboration between scientists, scientific institutions and governments. Funding through state resources and support from international organizations, such as the World Health Organization (WHO), also played a fundamental role (40).

Although the scientific society responded promptly by developing and approving novel vaccines and therapeutic agents against SARS-CoV-2, the global community faced deep inequalities in their access. For example, the European Union countries, including Greece, succeeded in achieving timely access to a sufficient amount of vaccine doses against SARS-CoV-2(2). The European Union prioritized the introduction of vaccination programs against SARS-CoV-2 as its principal political strategy against COVID-19 (https://health.ec.europa.eu/vaccination/overview_en). Developed countries gained privileged access to the first batches of vaccines, securing deals with pharmaceutical companies long before their release (41). The European Union countries responded quickly and prioritized solidarity in order to provide access to vaccines against SARS-CoV-2 to all the European citizens (https://commission.europa.eu/strategy-and-policy/coronavirus-response/coronavirus-european-solidarity-action_en). Moreover, international efforts, such as the global initiative COVAX, co-led by Gavi, the Vaccine Alliance, the Coalition for Epidemic Preparedness Innovations (CEPI), the WHO and UNICEF, sought to ensure equitable distribution of vaccines to developed and developing countries (41). However, all these efforts failed to meet their goals adequately. ‘Vaccine nationalism’ prominently affected the allocation of resources, as numerous governments chose to secure the needs of their populations neglecting international commitments. ‘Vaccine diplomacy’, as a political tool for foreign policy and international influence was also used as leverage to promote political and economic interests. These inequalities highlighted the gap between rich and poor countries and raised ethical and practical issues that may resonate and affect healthcare management of future crises.

The pandemic exposed the reciprocal relationship between politics and public health (42-44). Political leaders worldwide were challenged to make decisions that directly affected the spread of the virus, healthcare provision and the public perception of the pandemic. In numerous countries, decisions to impose lockdowns or lift restrictions were based on political calculations, such as the need to stabilize the economy or to respond to social pressure, rather than solely on scientific data and advice. The conflict between science and politics proved particularly harmful in cases where politicians downplayed the threat of the pandemic or spread misinformation, as witnessed in some countries with strong populist movements. These decisions undermined public trust in scientific authorities and challenged the implementation of necessary health measures. In several countries, political polarization and misinformation about vaccine safety increased vaccine hesitancy (45).

The need for updated training of healthcare professionals was another clear message from the recent COVID-19 pandemic (46,47). Political fora are expected to support the adjustment of medical educational programs to new realities and organize targeted actions involving the institutions responsible for providing ongoing medical education. Continuing medical education is critical as this could promote the value of medical education in paediatric viral infections as well, including COVID-19 (48,49). If the next pandemic disproportionally affects the paediatric population, this effort will play a key role for the preparedness of the paediatric personnel and healthcare system of each country.

For the post-COVID-19 era, long-term policies are required to prepare humanity for future health crises. The international community must ensure equal access to vaccines and therapeutic agents, regardless of the economic strength of a country. Governments need to collaborate with international organizations and the private sector to create a more resilient global public health system that can respond quickly and effectively to new threats and challenges (47). Our experience from the COVID-19 pandemic has taught us that health cannot be separated from politics and that protecting human life should be the highest priority, beyond economic or political calculations.

## 4. COVID-19 and science communication

Science communication-is a highly demanding process, which deals with complex information, dynamic uncertainty and diverse audiences, with varying educational levels, cultural beliefs, attitudes and behaviours, that impact the understanding of science (50). Effective science communication is now established as an important tool that provides accurate scientific knowledge to the public and helps them identify false information. Ineffective communication, on the other hand, can be detrimental to both science and society in general.

During the recent COVID-19 pandemic, science communication demonstrated its critical influence on public health. Since the beginning of the COVID-19 pandemic threat, healthcare professionals strived to translate science and communicate its ongoing findings in a timely and accessible manner to various audiences (51). Frontline researchers, alongside organizations such as the WHO, played the principal role to transparently communicate their findings and explain them to the public in a meaningful and understandable way. There was an unprecedented demand for scientific knowledge; in fact, the overwhelming requests from journalists for epidemiological and research updates threatened to shift focus and resources from viral research to media demands.

However, throughout the pandemic, non-specialist scientists, academics, journalists, and others played a key role in the communication of SARS-CoV-2 and COVID-19 advances, recommendations and challenges. Despite newspapers and press websites typically being reliable sources, the COVID-19 pandemic witnessed an unparalleled surge of both accurate and inaccurate information, largely spread via digital channels and platforms, such as Twitter/X, Facebook, Instagram and TikTok (52-54). Official scientific institutions and societies had to address issues, such as ‘fake news’ and uncertainty, the latter being a typical characteristic of scientific research, which was however misinterpreted and perceived as inaccuracy or even unreliability. Misinformation and disinformation regarding COVID-19 vaccine safety were strongly related to increased vaccine hesitancy (55-57).

Science communication with the aid of reliable and accessible official social media platforms was also encouraged (58). Innovative new communication strategies were proposed and used, including social media and podcasts (59). These tools were more effective at targeting specific audiences, such as adolescents and the young population. Science communication on COVID-19 pandemic required multidisciplinary scientific collaboration. Collaboration with visual communicators and design experts produced digital illustrations and demonstrations of SARS-CoV-2, which improved the understanding of the virus and health safety measures and improved vaccine confidence (60).

The post COVID-19 era offers a chance to assess the social impact of science communication and improve its future effectiveness. Researchers and scientific institutions need to design and develop novel communication strategies in order to respond effectively to future potential crises. Scientists with communication skills, passion and training should be motivated. Moreover, scientific societies should create improved links with the media and ensure that healthcare journalists are well informed and trained. Despite its devastating health, social, and financial ramifications, the COVID-19 pandemic presents a genuine opportunity to improve pandemic preparedness (61,62).

## 5. Conclusions

Pandemic evaluation and planning perspectives towards future infectious threats remain challenging. HFNOT, a non-invasive ventilation modality increasingly used prior to the COVID-19 era in both ward-based and critical care management of respiratory failure (11-25), represents an excellent clinical example of how the COVD-19 pandemic enriched medical knowledge and experience (26-32). The medical experience gained from the treatment of critically ill patients with COVID-19, should be further evaluated for the establishment of state-of-the-art, evidence-based medical consensuses and protocols. These tools are essential for the effective and precise management of adults and paediatric patients and should be integrated in current clinical practice.

The COVID-19 pandemic was a pivotal moment for global health and politics (33-47). The collaboration between science and politics contributed to the rapid development of vaccines and therapeutic agents against SARS-CoV-2, however global distribution was uneven due to national policies and geopolitical tensions. Different political agendas influenced not only the distribution of vaccines but also the public perception of their safety and efficacy. Therefore, the international community must be taught from these errors and work towards a more equitable and resilient approach to future health crises. Public health safety necessitates collaboration and impartiality, prioritizing global solidarity and equality above national and political agendas. Political decisions focusing on increasing the financial health resources in primary health care and advancing secondary and tertiary hospital-based care should be encouraged. Health policies should also focus on enhancing specialized as well as continuing medical education (46-49).

Science communication also demonstrated its potential usefulness and effectiveness during the recent pandemic (50-62). This burgeoning scientific discipline should be further developed and integrated into both undergraduate and postgraduate medical education. Health professionals must develop effective communication skills and become adept in providing accurate and useful information to their patients and the general population, as well. In the unfortunate event of a future pandemic, effective science communication will depend on multi-disciplinary collaboration between clinical and research scientists and communication experts; this task requires improved digital tools and innovative strategies to address public misinformation and disinformation.

The aim of the present review article was to stimulate further discussion within the international scientific community on the evaluation of the management of the recent COVID-19 pandemic. A careful interpretation of the lessons learned may help promote strategic planning and preparedness, and advance public health, translational research and future medicine.

## Figures and Tables

**Table I:** Key lessons from the COVID-19 pandemic: The role of intensive care, politics and science communication.

Topic	Main points and take-home messages (Refs.)
COVID-19 and intensive care medicine	HFNOT is a relatively novel technique for delivering warm, humidified oxygen at high flow rates to patients with acute hypoxaemic respiratory failure, including COVID-19 cases (11-32).
	HFNOT is supported by strong physiological evidence; HFNOT has been shown to improve oxygenation, reduce the work of breathing and enhance lung function (12-15).
	During the recent COVID-19 pandemic, HFNOT has been increasingly used in ICU and PICU patients due to its effectiveness and particularly its tolerability (27-32).
	Meta-analyses on the use of HFNOT in acute hypoxaemic respiratory failure and COVID-19 infection have generally shown that HFNOT is more effective than COT in reducing the need for higher respiratory support and comparable to NIV (27-30).
	HFNOT can be applied in the early management of hypoxemia and respiratory distress in children admitted to the PICU (23-26,31,32).
COVID-19 and politics	The COVID-19 pandemic highlighted the deep interactions between science, society, and politics; the rapid development of vaccines was a significant scientific achievement, driven by the collaboration of scientists, governments and international organizations (33-47).
	Despite various international efforts, inequalities in vaccine access underscored the economic and geopolitical aspects of the COVID-19 pandemic (41).
	Politics played a crucial role in managing the pandemic, with decisions regarding lockdowns and vaccination programs often influenced by political calculations, such as economic stability and social pressure, rather than solely by scientific data (33-47).
	The conflict between science and politics, as well as misinformation, undermined public trust and complicated the implementation of necessary measures (44).
	The need for political focus on the updated training of healthcare professionals was another key message from the recent COVID-19 pandemic (45-47).
COVID-19 and science communication	During the COVID-19 pandemic, frontline researchers around the globe, along with their official institutions and scientific societies, had the principal role in transparently communicating information about SARS-CoV-2 to the public (50-62).
	However, throughout the pandemic, non-specialist scientists and others also assumed a key role in public communication, while an unprecedented surge of information, disinformation and misinfor- mation about SARS-CoV-2 was spread especially via the social media platforms (52-54).
	Science communication on COVID-19 was another example that required multidisciplinary collaboration with communication experts leading to the urgent development and usage of innovative communication strategies (50-62).
	The post-COVID-19 pandemic period provides a valuable opportunity to evaluate the relationship between science communication and society to improve the preparedness of the international scientific community for the next pandemic (61,62).

COVID-19, coronavirus disease 2019; HFNOT, high flow nasal oxygen therapy; ICU, intensive care unit, PICU, paediatric intensive care unit; COT, conventional oxygen therapy; NIV, non-invasive ventilation; SARS-CoV-2, severe acute respiratory syndrome coronavirus 2.

## Data Availability

Not applicable.
